# Novel mutation in *KCNJ2* gene causes long QT interval syndrome type 7 and learning disability: A case report

**DOI:** 10.1097/MD.0000000000041056

**Published:** 2024-12-27

**Authors:** Hua-yong Zhang, Yong Zhang

**Affiliations:** a Department of Cardiology, Wuhan Children’s Hospital (Wuhan Maternal and Child Healthcare Hospital), Tongji Medical College, Huazhong University of Science & Technology, Wuhan, China; b Clinical Medical Research Center for Birth Defect Prevention and Treatment in Wuhan, China.

**Keywords:** case report, inward-rectifier potassium ion channel, *KCNJ2* gene, learning disability, long QT interval syndrome type 7

## Abstract

**Rationale:**

Long QT interval syndrome type 7 (LQT7) is a rare hereditary multisystem disorder characterized by a classic triad of ventricular arrhythmias with QT interval prolongation, periodic paralysis, and distinctive skeletal and facial features. The Kir2.1 protein is encoded by the *KCNJ2* gene, which has been associated with LQT7.

**Patient concerns:**

We report an 8-year-old boy who presented with frequent premature ventricular contraction with QRS electrical alternans, QT interval prolongation, bidirectional ventricular tachycardia, and learning disability with poor school performance. Gene sequencing revealed a novel missense mutation in the *KCNJ2* gene (c.224 C>A, p.Thr75Lys).

**Diagnoses:**

The patient was diagnosed as LQT7 and a learning disability.

**Interventions:**

During the follow-up period, the ventricular arrhythmias were difficult to treat with β-blocker. Due to the frequent premature ventricular contraction and bidirectional ventricular tachycardia, radiofrequency catheter ablation was tried but failed.

**Outcomes:**

An implantable cardioverter-defibrillator was recommended due to the recurrent syncope, but the boy’s legal guardian rejected the recommendation, opting to continue his treatment in another hospital.

**Lessons:**

Clinical management is mostly focused on reducing adverse cardiac events. As a first option, β-blockers are often chosen as treatments for LQT7 patients, but there is no clear evidence for their effectiveness in preventing fatal arrhythmias. If the drug treatment is not effective, radiofrequency catheter ablation can be considered. However, it may be difficult to target accurately the right spot, and the attempt of the radiofrequency catheter ablation failed. Therefore, after ineffective medical treatment, implantable cardioverter-defibrillator implantation could be an option for patients with life-threatening cardiac events.

## 1. Introduction

Long QT interval syndrome type 7 (LQT7), also known as Anderson–Tawil syndrome, is a rare hereditary multisystem disorder characterized by a classic triad of ventricular arrhythmias with QT interval prolongation, periodic paralysis, and distinctive skeletal and facial features. However, considerable inter-individual variability exists in clinical manifestations. Only partial clinical features were displayed in some patients. The first description of LQT7 was in 1963, with an estimated prevalence of <1/1,000,000.^[1–3]^ Mutations in the inward-rectifier potassium channel protein Kir2.1 on chromosome 17q24.3 cause LQT7 type-1. In contrast, patients with unknown mutations are designated as LQT7 type-2. A mutation in the KCNJ5 gene on chromosome 11q24.3 encoding a G-protein-activated inward-rectifier potassium channel protein, Kir3.4, has been reported to cause a similar phenotype.^[1,4]^

There is a high expression of Kir2.1 in the skeletal and cardiac muscles, as well as in the brain. In the human heart, a dysfunctional Kir2.1 channel prolongs phase 3 of the potential, which is characterized by a prolonged QT interval, pathologic U waves, and a special type of polymorphic ventricular tachycardia (VT) called bidirectional ventricular tachycardia (BiVT) in the electrocardiogram.^[5]^ It is not only the heart that expresses Kir2.1, but also the brain. Therefore, the neurological or neurocognitive symptoms of patients with *KCNJ2* gene mutation, including mild learning difficulties, problems with memory or concentration, psychological problems, and depression would be conceivable.^[1]^ Here, we report a young boy with LQT7 and a learning disability due to a de novo missense mutation in the *KCNJ2* gene (c.224 C > A, p.Thr75Lys).

## 2. Case presentation

An 8-year-old boy with a 2-year history of cardiac arrhythmias was referred to our hospital in April 2021. Two years ago, he had a history of symptomatic syncope experience in the early morning. The symptoms included loss of consciousness, cyanotic lips, pale complexion, cold limbs, and incontinence of urine, lasting for 1~2 minutes. For further analysis, he was transferred to our hospital. Standard 12-lead electrocardiogram and 24-hour Holter monitoring revealed paroxysmal VT, frequent premature ventricular contraction (PVC), and the QT interval prolongation (QTc interval > 480 ms). Blood examinations, echocardiogram, brain magnetic resonance imaging, and electroencephalogram showed no abnormalities. The initial diagnosis of LQT syndrome was established. Subsequently, genetic testing was requested after obtaining informed consent from his parents and older sister. Concurrently, oral propranolol was administered to decrease the risk of cardiac adverse events. During the follow-up period of 2 years, no syncope event or episode of periodical paralysis occurred. However, these ventricular arrhythmias were difficult to treat with propranolol.

On physical examination, his heart rate was 84 beats/minute and irregular, blood pressure 108/58 mm Hg, height 131 cm (*P*_25th_*~P*_50th_), and weight 24.5 kg (*P*_10th_*~P*_25th_). The dental malalignment and diastema were noted, and the remainder of the physical examination was normal.

In addition, he had a learning disability with poor school performance. The intelligence quotient was 81 evaluated by the Wechsler Intelligence Scale. Family history revealed no specific abnormalities.

**Figure 1.**
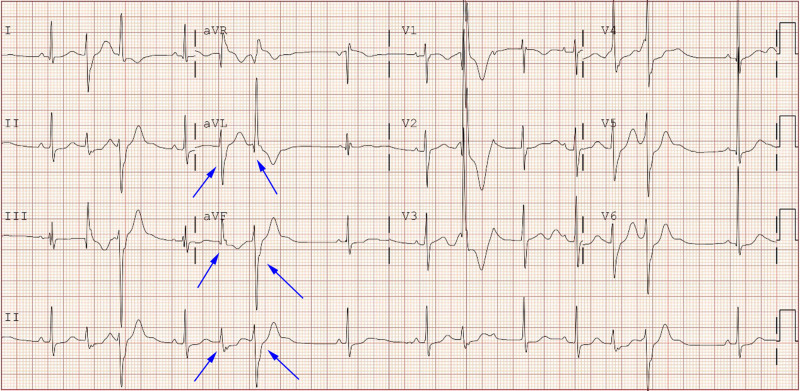
Standard 12-lead electrocardiogram showed frequent premature ventricular contraction with QRS electrical alternans (blue arrow).

**Figure 2.**
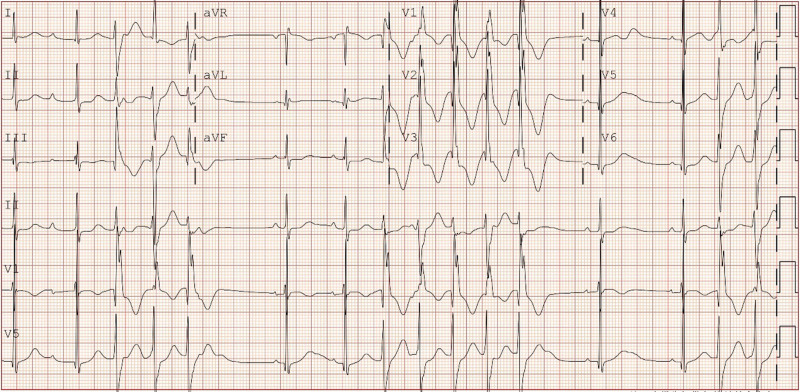
Standard 12-lead electrocardiogram showed left posterior fascicular ventricular tachycardia.

**Figure 3.**
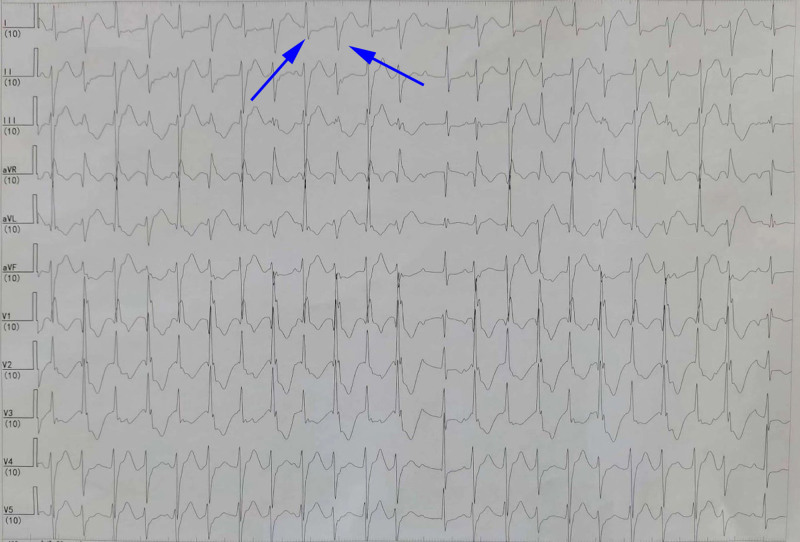
A 24-hour Holter monitoring showed polymorphic ventricular tachycardia called bidirectional ventricular tachycardia (blue arrow).

A 12-lead electrocardiogram and 24-hour Holter monitoring were reexamined after admission to the hospital, which showed frequent PVC with QRS electrical alternans (showed in Fig. 1), QTc interval > 480 ms, left posterior fascicular VT (showed in Fig. 2), and polymorphic VT called BiVT (showed in Fig. 3). No abnormal changes in blood routine, blood biochemistry, echocardiographic examination, and cardiac magnetic resonance imaging were found.

**Figure 4.**
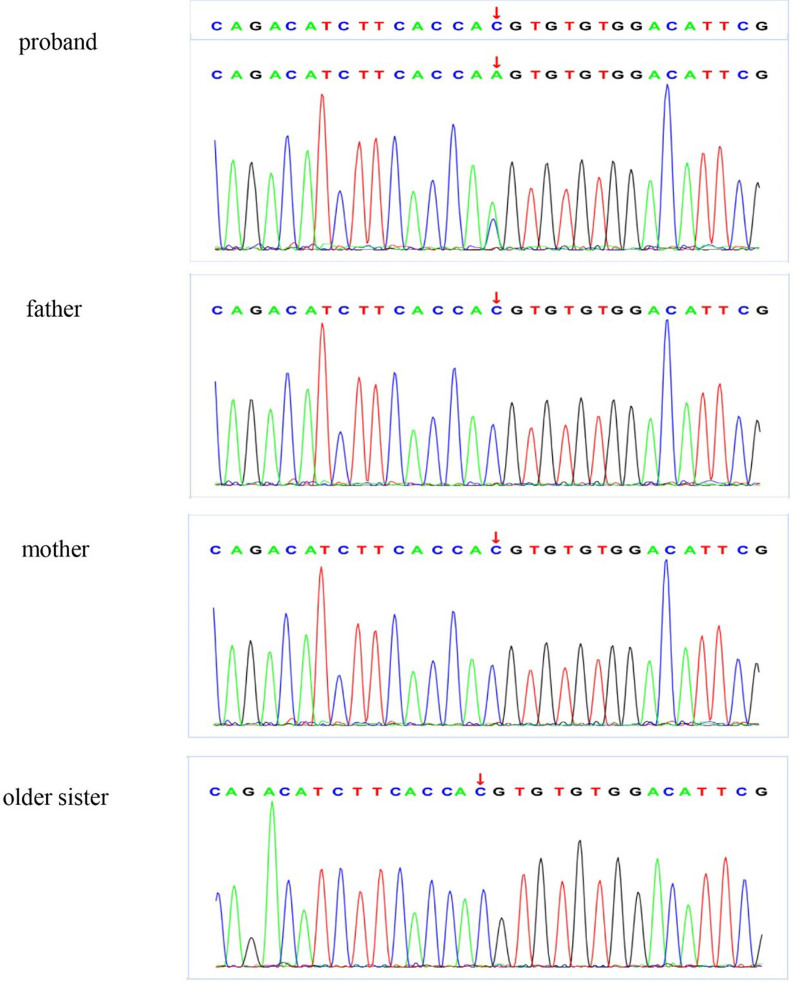
Whole-exome sequencing identified mutation in the proband. The identified point mutation in exon 2 of the KCNJ2, generated a novel missense mutation (c.224 C > A, p.Thr75Lys, NM_000891).

Here, we reviewed the previous results of whole-exome sequencing (WES) using peripheral blood genomic DNA from the patient, his older sister, and his parents. A novel missense mutation c.224 C > A (p.Thr75Lys) was found in the *KCNJ2* gene of the proband, which was thought to be causal to LQT7 (shown in Fig. 4). The variant is located in exon 2, which leads to the replacement of threonine by lysine at position 75 in the protein sequence. It was not previously recorded in the general public databases of gnomAD database, 1000 gene database, dbSNP, or previous literature. This variant affects a highly conserved amino acid located in the amino terminus of the Kir2.1 channel and is predicted to be deleterious by the protein function prediction software Polyphen2, SIFT, and Mutation Taster. American College of Medical Genetics and Genomics (ACMG) guidelines and standards describe this variant as pathogenic.

Fully informed consent was obtained from the patient and his parents. Radiofrequency catheter ablation targeting the frequent PVC and VT was subsequently prepared to undergo. However, possibly due to general anesthesia, any spontaneous arrhythmias were not demonstrated leading to the failure of preoperative electrophysiological mapping and radiofrequency ablation treatment. Due to only 1 episode of syncope, his parents declined the implantation of an implantable cardioverter-defibrillator (ICD). Furthermore, the patients may place low trust in doctors, and therefore they were not reluctant to try other antiarrhythmic drugs including flecainide and verapamil. During the follow-up period of 1 year, the episode of syncope did recur. ICD was recommended, but the patient’s legal guardian again rejected the recommendation, opting to continue his treatment in another hospital (the timeline showed in Fig. S1, Supplemental Digital Content, http://links.lww.com/MD/O239).

## 3. Discussion

There is a rare autosomal dominant disorder called LQT7 which is characterized by ventricular arrhythmias, periodic paralysis, and distinctive skeletal and facial features.^[1]^ In this case report, we identified a novel missense mutation of the *KCNJ2* gene (NM_000891: c.224C > A, p.Thr75Lys) causing LQT7 and learning disability, without periodic paralysis, or distinctive skeletal and facial features.

KCNJ2 encodes the inward-rectifier potassium ion channel protein Kir2.1, which regulates resting membrane potentials in the heart, skeletal muscle, and brain.^[6]^ In the human heart, Kir2.1 is abundantly expressed in the ventricle, but less so in the atrium.^[1]^ Kir2.1 is a component of the inward rectifier current IK1, which is activated after the plateau phase and provides the dominant current in the late repolarization phase of the cardiac action potential.^[6]^ A Thr75Lys mutation replaces a highly conserved threonine residue in the slide helix near the cytoplasmic N-terminus.^[1,6–8]^ The high degree of conservation of Thr75 suggests that the mutation has an important consequence on the Kir2.1 function. Previous studies showed that the mutations in the same locus of the *KCNJ2* gene (T75A, T75M, and T75R) did not affect the protein expression of Kir2.1, but impaired membrane localization of the channel protein, which led to the loss-of-function of Kir2.1 and possessed a strong dominant negative effect.^[6–8]^

The clinical rarity and wide range of phenotypic characteristics of LQT7 make diagnosis challenging. Only 58%~78% of LQT7 patients present with all triad symptoms.^[3]^ Additionally, different genes lead to the same clinical phenotypes, while the same genetic error can result in different clinical phenotypes. Approximately 60% to 70% of LQT7 have causal mutations in KCNJ2, and the mutations in KCNJ5 can also cause a similar phenotype of LQT7.^[1,4]^ The loss-of-function mutations in KCNJ2 are associated with LQT7 and catecholaminergic polymorphic ventricular tachycardia, while the gain-of-function mutations can lead to the short QT syndrome type 3 and familial atrial fibrillation type 3.^[1,9,10]^ Even the phenotype attributed to the mutation of the same gene locus can also be inconsistent due to the effects of unidentified modifier loci.^[6–8]^ In our study, the proband had only ventricular arrhythmias with QT interval prolongation, without periodic paralysis and dysmorphic features, which showed frequent PVC with QRS electrical alternans, QTc interval > 480 ms, left posterior fascicular VT and polymorphic VT called BiVT. Radiofrequency catheter ablation treatment was tried but failed. During the follow-up period, ICD was recommended due to the recurrent syncope, but the patient’s legal guardian rejected the recommendation. Furthermore, the proband had a learning disability with poor school performance. The intelligence quotient was 81 evaluated by the Wechsler Intelligence Scale. Previous research had not revealed much information about psychological or cognitive complications among patients with LQT7, which would be valued due to the mutation expressed not only in the heart, and skeletal muscle but also in the brain.^[1,11]^ Slight cognitive defects had been noticed, including mild learning difficulties, problems with memory or concentration, psychological problems, and medication for depression.^[1,12,13]^ Indeed, some scholars believe that the Gly300Asp mutation may lead to mild learning difficulties.^[12]^

Currently, clinical management is mostly focused on reducing adverse cardiac events. As a first option, β-blockers are often chosen as treatments for LQT7 patients, but there is no clear evidence for their effectiveness in preventing fatal arrhythmias.^[1,14]^ Therefore, it is not surprising that treatment with β-blockers is often ineffective.^[1]^ Still, previous studies have concluded that ventricular arrhythmias can be controlled by oral flecainide treatment or the combination treatment of β-blocker and flecainide.^[15–18]^ However, the exact molecular mechanism underlying the effectiveness of flecainide is not clear.^[1]^ Moreover, the calcium channel inhibitor verapamil has also been used to suppress BiVT, decrease the frequency of PVC, and unmask a prolonged QT interval.^[1]^ As a general rule and precaution, the use of QT-prolonging drugs should be avoided to prevent life-threatening cardiac events. If the drug treatment is not effective, radiofrequency catheter ablation can be considered. However, it may be difficult to target accurately the right spot, and the attempt of the radiofrequency catheter ablation failed.^[19,20]^ Therefore, after ineffective medical treatment, ICD implantation could be an option for patients with life-threatening cardiac events.^[1,21]^

In conclusion, we identified a novel missense mutation of the *KCNJ2* gene (c.224 C > A, p.Thr75Lys), leading to frequent PVC with QRS electrical alternans, QT interval prolongation, BiVT, and learning disability. The clinical findings expanded the genotype-phenotype correlation.

## 4. Patient perspective

LQT7 is a rare hereditary multisystem disorder. Approximately 60% to 70% of LQT7 is caused by KCNJ2 mutations. It is challenging to diagnose LQT7 early due to the clinical rarity and high degree of phenotypic variability.

Clinical management is mostly focused on reducing adverse cardiac events. Individualized and targeted therapies, including flecainide or the combination treatment of β-blocker and flecainide or ICD implantation, have been reported to reduce the life-threatening cardiac events for LQT7 patients significantly. Due to lacking trust, they declined the individual optimum therapy options, which greatly increased the risk of adverse cardiac events.

## Author contributions

**Conceptualization:** Yong Zhang.

**Data curation:** Hua-yong Zhang.

**Funding acquisition:** Hua-yong Zhang.

**Investigation:** Hua-yong Zhang, Yong Zhang.

**Methodology:** Hua-yong Zhang, Yong Zhang.

**Project administration:** Hua-yong Zhang, Yong Zhang.

**Resources:** Yong Zhang.

**Software:** Hua-yong Zhang.

**Supervision:** Yong Zhang.

**Visualization:** Hua-yong Zhang.

**Writing – original draft:** Hua-yong Zhang.

**Writing – review & editing:** Yong Zhang.

## Supplementary Material


