# Supplementary figures and images for: Novel mutation in KCNJ2 gene causes long QT interval syndrome type 7 and learning disability: A case report
Source: Medicine (Baltimore). 2024 Dec 27;103(52):e41056. doi: 10.1097/MD.0000000000041056 (PMC11688009; doi:10.1097/MD.0000000000041056)

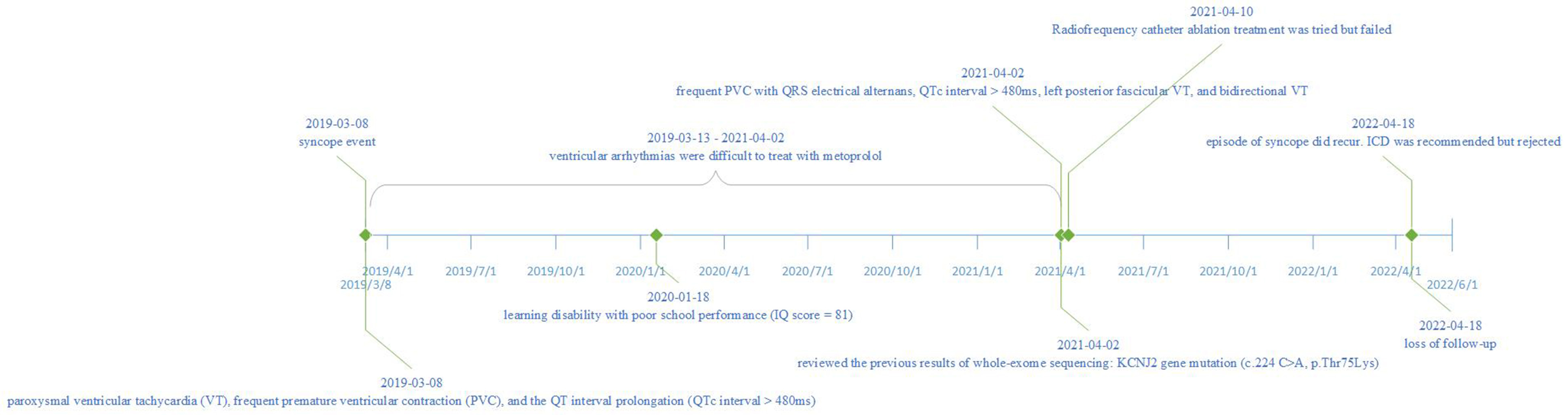

Supplement: Supplementary file 1 [file medi-103-e41056-s001.tif]
